# Preventive efficacy of four or six monthly oral doses of 24 µg/kg moxidectin compared to six monthly doses of Heartgard® Plus or Interceptor® Plus against macrocyclic lactone-resistant heartworm (*Dirofilaria immitis*) strains in dogs

**DOI:** 10.1186/s13071-020-04178-z

**Published:** 2020-07-14

**Authors:** Kristina Kryda, Susan Holzmer, William R. Everett, John W. McCall, Sean P. Mahabir, Tom L. McTier, Steven J. Maeder

**Affiliations:** 1 Zoetis, Veterinary Medicine Research and Development, 333 Portage St, Kalamazoo, MI 49007 USA; 2 Bertek Inc, 104 Wilson Bottoms Rd, Greenbrier, AR 72058 USA; 3 TRS Labs Inc, 215 Paradise Blvd, Athens, GA 30607 USA

**Keywords:** Canine, *Dirofilaria immitis*, Heartgard® Plus, Heartworm, Interceptor® Plus, Laboratory study, Macrocyclic lactone, Moxidectin, Resistance

## Abstract

**Background:**

Recent reports indicated that increasing the monthly oral dosage and the number of consecutive monthly doses of moxidectin improved the efficacy against macrocyclic lactone (ML)-resistant *Dirofilaria immitis*. The two laboratory studies reported here evaluated the efficacy of four or six monthly oral doses of 24 µg/kg moxidectin compared to six monthly doses of either Heartgard® Plus (ivermectin/pyrantel) or Interceptor® Plus (milbemycin oxime/praziquantel) against ML-resistant *D. immitis* strains.

**Methods:**

Dogs were inoculated 30 days prior to first treatment with 50 third-stage (L_3_) larvae of a ML-resistant strain of *D. immitis*, ZoeLA or JYD-34. In each study, dogs (six per group) were randomized to treatment with six monthly doses of placebo, four or six monthly doses of 24 µg/kg moxidectin, or six monthly doses of Heartgard® Plus or Interceptor® Plus at their label dose rates. Efficacy was evaluated by adult heartworm counts approximately nine months after L_3_ inoculation.

**Results:**

All negative-control dogs were infected with adult heartworms (geometric mean, 35.6; range, 24–41) for ZoeLA and (geometric mean, 32.9; range, 30–37) for JYD-34. Efficacies against ZoeLA for moxidectin, Heartgard® Plus and Interceptor® Plus were ≥ 96.1%, 18.7% and 21.2%, respectively. Adult counts for both moxidectin-treated groups were significantly lower than negative control (*P *< 0.0001), significantly lower than Heartgard® Plus and Interceptor® Plus (*P *< 0.0001), but not significantly different from each other (*P *= 0.5876). Counts for Heartgard® Plus and Interceptor® Plus were not significantly different than negative control (*P* ≥ 0.2471). Efficacies against JYD-34 were ≥ 95.9%, 63.9% and 54.6% for moxidectin, Heartgard® Plus and Interceptor® Plus, respectively. Counts for all groups were significantly lower than negative control (*P* ≤ 0.0001). Counts for six monthly doses of moxidectin were significantly lower than those for four monthly doses (*P *= 0.0470), and the counts for both moxidectin-treated groups were significantly lower than Heartgard® Plus and Interceptor® Plus (*P* ≤ 0.0002).

**Conclusions:**

Moxidectin administered orally at 24 µg/kg to dogs for four or six consecutive months was ≥ 95.9% effective in preventing the development of two ML-resistant heartworm strains and resulted in significantly fewer adult *D. immitis* than in dogs treated with Heartgard® Plus or Interceptor® Plus when administered for six consecutive months at their approved label dosages in two laboratory efficacy studies.
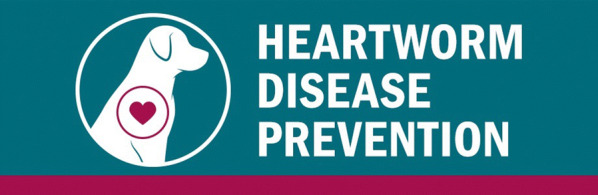

## Background

Heartworm disease in dogs is caused by the mosquito-borne parasitic nematode *Dirofilaria immitis.* Adult *D. immitis* reside in the dogs’ pulmonary arteries and heart, and infections can cause severe cardiopulmonary disease which can be life-threatening.

Canine heartworm disease is widespread in many parts of the world, with higher prevalence in warmer climates which best support the more than 60 different mosquito vectors [1]. Geographical distribution appears to be expanding, potentially due to changes in climate, increasing pet travel, and the expansion of mosquito vectors into new areas [2, 3].

The macrocyclic lactone (ML) class of anthelmintics are the only drug class widely available as heartworm preventatives and are highly effective in the prevention of canine heartworm disease [4]. These drugs work, likely in concert with the dog’s immune system, by killing the immature third (L_3_) and fourth (L_4_) larval stages of *D. immitis* thus preventing development to adult worms [5]. Lack of efficacy (LOE) of ML heartworm preventatives can result from failure to regularly administer the product according to the approved label directions [4]; however, lack of efficacy due to *D. immitis* strains resistant to MLs is a growing concern [4].

Moxidectin is a ML that is available in heartworm preventative products worldwide with 100% efficacy reported against ML-susceptible *D. immitis* strains [4]. Moxidectin has unique attributes that make it an attractive molecule for use as a heartworm preventative in the face of emerging resistance [6]. Recent reports showed that increasing the oral monthly dosage of moxidectin and the number of consecutive monthly doses administered improved its efficacy against ML-resistant *D*. *immitis*, and that 24 µg/kg was selected as the optimal dosage for further commercial development [4].

Here we present two studies that compare the preventive efficacy of four or six monthly doses of moxidectin administered orally at 24 µg/kg to six monthly doses of Heartgard® Plus (ivermectin/pyrantel) or Interceptor® Plus (milbemycin oxime/praziquantel) administered orally at their approved label dose rates against two confirmed ML-resistant *D. immitis* strains.

## Methods

### Design

These two negative-controlled, comparative, masked, randomized laboratory studies were conducted according to the International Co-operation on Harmonization of Technical Requirements for Registration of Veterinary Medicinal Products (VICH) GL7, “Efficacy of Anthelmintics: General Requirements” [7], VICH GL19 “Efficacy of Anthelmintics: Specific Recommendations for Canines” [8], and complied with VICH GL9 “Good Clinical Practice” [9].

Personnel involved in making assessments of efficacy or safety, including *D. immitis* L_3_ inoculation, treatment administration, physical examination, clinical observation, and necropsy and adult heartworm recovery were masked to treatment assignments.

Study 1 evaluated efficacy against the ML-resistant *D. immitis* ZoeLA strain, and Study 2 evaluated efficacy against the ML-resistant *D. immitis* JYD-34 strain. Both studies were conducted using the same design, which is summarized in Table 1.

**Table 1:** Efficacy of oral moxidectin against ML-resistant heartworms compared to Heartgard® Plus or Interceptor® Plus: study designs

Group	Treatment	Oral dosage	No. of dogs	Day of L_3_*D. immitis* inoculation^a^	Days of treatment	Days of blood microfilariae and adult *D. immitis* antigen testing^b^	Day of necropsy and adult *D. immitis* worm recovery
T01	Negative control	na	6	-30	0, 30, 60, 90, 120, 150	-36 or -30, 60, 180, 210, 236 or 243	237 or 243
T02	Moxidectin	24 µg/kg	6	-30	0, 30, 60, 90		
T03	Moxidectin	24 µg/kg	6	-30	0, 30, 60, 90, 120, 150		
T04	Heartgard® Plus	Min. of 6 µg/kg^c^	6	-30	0, 30, 60, 90, 120, 150		
T05	Interceptor® Plus	Min. of 500 µg/kg^c^	6	-30	0, 30, 60, 90, 120, 150		

^a^Each dog was inoculated with 50 infective L_3_ of *D. immitis* of either ZoeLA (Study 1) or JYD-34 (Study 2)

^b^Day 180 testing only conducted in Study 2

^c^Heartgard® Plus and Interceptor® Plus administered at their approved dosages according to their commercial label directions

### Animals

Six dogs, both males and females, were included in each treatment group. Dogs were individually identified by ear tattoo or microchip and were purpose-bred laboratory beagles or mixed breeds. At the time of *D. immitis* L_3_ inoculation, dogs ranged in age from two to three months in Study 1, and from four to five months in Study 2. At the time of the first treatment administration, body weight ranged from 3.6 to 9.0 kg in Study 1, and from 6.3 to 9.1 kg in Study 2. All dogs were assessed as being in good health at the time of enrollment based on physical examination by a veterinarian. All dogs were negative for blood microfilariae and adult *D. immitis* antigen prior to L_3_ inoculation. None of the dogs had ever received ProHeart® 6 (moxidectin; Zoetis, Parsippany, NJ, USA) or any ML-containing product within 90 days prior to the start of the study.

Dogs were housed indoors with two dogs in each pen within a mosquito-proof facility in cages that complied with accepted animal welfare legislation and guidance. Standard environmental conditions were maintained, and environmental enrichment and social interactions were provided. Dogs were fed an appropriate commercial canine maintenance diet and had access to water *ad libitum*. Dogs were acclimated at the facility for 7 days prior to L_3_ inoculation and were observed for general health at least once daily throughout the studies.

### *Dirofilaria immitis* strains

The *D. immitis* strains used in these studies were derived from isolates collected from naturally infected dogs located in the middle to southeastern USA. ZoeLA (Study 1) was collected from a dog in Louisiana in June of 2013, and JYD-34 (Study 2) was collected from a dog in Illinois in July of 2010. Both isolates were validated as infective strains through the diagnosis of circulating microfilariae, positive heartworm antigen test results, and adult worm recovery in recipient animals following inoculation of L_3_ that had been derived directly from microfilariae obtained from the original field cases. Both ZoeLA and JYD-34 *D. immitis* strains have been demonstrated to be resistant to MLs [4, 10].

### *Dirofilaria immitis* L_3_ inoculations

The third-stage (L_3_) larvae used for inoculation were harvested from infected *Aedes aegypti* mosquitoes reared and maintained at Zoetis (Kalamazoo, MI, USA) using techniques previously described [11].

Thirty days prior to the first treatment administration on Day 0, each dog was administered 50 viable L_3_ by subcutaneous injection in the inguinal region, with the exception of one dog in the six month moxidectin group in Study 1 (efficacy against ZoeLA) which received 49 larvae. In this dog, one larva remained in the syringe after the initial injection and although the solution containing the larva was re-injected, the single larva remained in the syringe. Further attempts at re-injecting the remaining larva were not attempted.

### Randomization and treatments

Dogs were allocated to treatment (pen) according to a randomized complete block design with a one-way treatment structure. Dogs were ranked by Day-7 body weight within sex into blocks of 10 dogs, and within each block a pair of dogs were randomly allocated to treatment (pen).

Negative control dogs (T01) were administered an empty hydroxypropyl methylcellulose (HPMC) capsule on Days 0, 30, 60, 90, 120 and 150. Dogs treated with four monthly doses of moxidectin (T02) were administered moxidectin on Days 0, 30, 60 and 90, and in order to facilitate masking, were administered an empty HPMC capsule on Days 120 and 150. Dogs treated with six monthly doses of moxidectin (T03), Heartgard® Plus (T04), or Interceptor® Plus (T05) were administered the appropriate test material on Days 0, 30, 60, 90, 120 and 150.

Moxidectin was supplied as granules that were produced using a conventional wet granulation process. The granules contained moxidectin and excipients including a binder, bulking agent and disintegrant. The granules were placed in a HPMC capsule to deliver the exact calculated dose of moxidectin for each dog, therefore all moxidectin-treated dogs received exactly 24 µg/kg moxidectin. These granules were of the same composition as the moxidectin component of the chewable oral tablet product (sarolaner, moxidectin and pyrantel chewable tablets; Zoetis). Heartgard® Plus chewables and Interceptor® Plus chewable tablets were obtained from a commercial supplier and were administered according to their approved commercial dosing instructions, which resulted in dogs treated with Heartgard® Plus receiving 6.2–15.5 µg/kg ivermectin in Study 1 and 6.0–11.8 µg/kg ivermectin in Study 2, and dogs treated with Interceptor® Plus receiving 0.5–1.2 mg/kg milbemycin oxime in Study 1 and 0.5–1.0 mg/kg milbemycin oxime in Study 2.

Body weights obtained within the 7 days prior to each dose administration were used for dose calculation. Feed was withheld overnight prior to treatment administration and was not offered again until at least 2 h after treatment administration. All doses were administered by mouth and each dog was observed for several minutes after dosing for evidence that the dose was swallowed, and for approximately 2 h after dosing for evidence of emesis. Except for the Day 0 dosing in Study 1 (efficacy against ZoeLA), dogs were housed individually for the post-dose observation period.

### Blood microfilariae and adult *D. immitis* antigen testing

Blood was collected from each dog on Days -36 or -30, 60, 180 (only in Study 2; efficacy against JYD-34), 210, and 236 or 243 for examination for blood microfilariae and adult *D. immitis* antigen testing. Testing of blood collected on Days -36 or -30 and 60 was conducted to detect *D. immitis* infections that may have been present prior to experimental inoculation, and testing of samples collected on Days 180, 210, and 236 or 243 was conducted to detect infection from the experimental *D. immitis* inoculation.

A modified Knott’s procedure was used for the blood microfilariae examination and a commercially available test kit (DiroCHEK® Heartworm Antigen Test Kit; Zoetis) was used for detection of *D. immitis* antigen [5]. Any samples negative for *D. immitis* antigen on Day 236 or 243 were heat-treated and the antigen testing repeated.

### Health observations

General health observations were made for each dog twice daily except when physical examinations were performed (once within the 6 days prior to L_3_ inoculation and prior to euthanasia) and on days of treatment administration. Directed clinical observations were made on all dogs prior to and at 1, 3, 6 and 24 h after each treatment administration.

### Necropsy and adult *D. immitis* worm recovery

On Day 237 (Study 2) or 243 (Study 1), all dogs were humanely euthanized with a pentobarbital euthanasia solution administered according to the approved label directions in addition to added heparin given intravenously. The pleural and peritoneal cavities were examined for adult *D. immitis*, the posterior and anterior vena cavae were clamped, and the heart and lungs were removed. The precava, right atrium, right ventricle and pulmonary arteries (including those coursing through the lungs) were dissected and examined. Any adult worms present were recovered and classified as male or female and as either dead (worms abnormal in both appearance and motility) or alive (all other worms) as previously described [12]. Dogs were randomly assigned to order of euthanasia and necropsy.

### Statistical analysis

The experimental unit for treatment was the pen, and the primary endpoint was the total (live + dead) worm count. Worm counts were transformed by the ln (count + 1) transformation prior to analysis in order to stabilize the variance and normalize the data. Transformed counts were analyzed using a mixed linear model (SAS 9.4, Cary, NC, USA) that included the fixed effect of treatment, and the random effects of block, interaction between block and treatment (pen term), and error. Testing was two-sided at the significance level α= 0.05.

Percent efficacy relative to negative control was calculated using geometric means (back-transformed least square means) based on the formula [(C − T)/C] × 100, where C is the mean worm count for the placebo group and T is the mean worm count for the treated group.

## Results

Dosing was uneventful and no capsules, chews, or chewable tablets were expelled during or after dosing, and no emesis of capsules, chews, or chewable tablets was noted during post-treatment observations with the exception of two events during treatment on Day 0 and one event on Day 30 in Study 1 (efficacy against ZoeLA): on Day 0, one Heartgard® Plus-treated dog was observed to vomit an intact chew approximately 1 h after dosing; the chew was re-administered uneventfully. Also, on Day 0, approximately 30 min following dosing, an intact capsule was found beneath a cage that housed 2 of the 6 dogs allocated to receive four monthly doses of moxidectin. Because the capsule was expelled after the planned post-dose observation period, the event was not observed, and therefore it was not possible to determine which of the two dogs had expelled the capsule. In order to avoid over-dosing of the dog that did not expel its capsule, the capsule found under the cage was not re-administered to either dog. As a result, one of these two dogs did not receive its full intended Day 0 treatment and therefore only received three complete monthly doses of moxidectin, while the remaining dog received 4 complete monthly doses according to the study design.

This mis-dosing event did not have any significant impact on the study results. Adult *D. immitis* worm counts for the group treated with four monthly doses of moxidectin ranged from 0 to 3, and the worm counts for the two dogs involved in the event were similar with 2 worms recovered from one dog and 3 worms recovered from the other dog.

Following this event, all dogs in both studies were housed individually for the post-dose observation period so that any expelled capsules could be positively attributed to an individual dog. On Day 30, a whole chew was discovered approximately 20 min after dosing of one Heartgard® Plus-treated dog; the chew was successfully re-administered.

There were no mortalities and no treatment-related adverse reactions in either study. Observations of abnormal health were minor, and the incidences of occurrence were generally similar between treatment groups. Abnormal health events were of the type commonly observed in laboratory dogs, and included gastrointestinal, dermatologic, ophthalmic, otic, orthopedic, urinary tract and musculoskeletal abnormalities.

### Efficacy against the ML-resistant ZoeLA strain

Blood microfilariae and adult *D. immitis* antigen results are summarized in Table 2. Adult *D. immitis* worm counts, efficacies relative to negative control, and statistical comparisons are summarized in Table 3.

**Table 2:** Adult *D. immitis* antigen and microfilariae test results (ZoeLA strain)

Treatment group	Oral dosage	Days of treatment	Individual	Day -36		Day 60		Day 210		Day 243	
				Ag	MF	Ag	MF	Ag	MF	Ag	MF
Negative control	na	0, 30, 60, 90, 120, 150	1	−	−	−	−	+	+	+	+
			2	−	−	−	−	+	+	+	+
			3	−	−	−	−	+	+	+	+
			4	−	−	−	−	+	+	+	+
			5	−	−	−	−	+	+	+	+
			6	−	−	−	−	+	+	+	+
Moxidectin	24 µg/kg	0, 30, 60, 90	1	−	−	−	−	−	+	−	−
			2	−	−	−	−	+	−	+	−
			3	−	−	−	−	−	−	−	−
			4	−	−	−	−	−	−	+	−
			5	−	−	−	−	+	−	+	−
			6	−	−	−	−	+	+	+	+
Moxidectin	24 µg/kg	0, 30, 60, 90, 120, 150	1	−	−	−	−	−	+	−	−
			2	−	−	−	−	−	−	−	−
			3	−	−	−	−	−	+	−	−
			4	−	−	−	−	−	−	−	−
			5	−	−	−	−	−	−	−	−
			6	−	−	−	−	−	−	−	−
Heartgard® Plus	Min. of 6 µg/kg^a^	0, 30, 60, 90, 120, 150	1	−	−	−	−	+	+	+	+
			2	−	−	−	−	+	+	+	+
			3	−	−	−	−	+	+	+	+
			4	−	−	−	−	+	+	+	+
			5	−	−	−	−	+	+	+	+
			6	−	−	−	−	+	−	+	−
Interceptor® Plus	Min. of 500 µg/kg^b^	0, 30, 60, 90, 120, 150	1	−	−	−	−	+	+	+	+
			2	−	−	−	−	+	+	+	+
			3	−	−	−	−	+	+	+	+
			4	−	−	−	−	+	+	+	−
			5	−	−	−	−	+	+	+	+
			6	−	−	−	−	+	+	+	+

^a^Heartgard® Plus (ivermectin + pyrantel pamoate) administered according to commercial label directions which resulted in administration of 6.2–15.5 µg/kg of ivermectin

^b^Interceptor® Plus (milbemycin oxime + praziquantel) administered according to commercial label directions which resulted in administration of 0.5–1.2 mg/kg milbemycin oxime

*Abbreviations*: Ag, adult *D. immitis* antigen test; MF, *D. immitis* microfilariae test; −, negative; +, positive

**Table 3:** Efficacy of oral moxidectin (24 µg/kg) compared to Heartgard® Plus or Interceptor® Plus (ZoeLA strain)

Treatment	Oral dosage	Days of treatment	No. of infected dogs^a^	Adult *D. immitis* worm counts		Efficacy compared to negative control^c^
				Individual worm counts	Geometric mean^b^	% Reduction
Negative control	na	0, 30, 60, 90, 120, 150	6 of 6	24, 36, 36, 39, 41, 41	35.6^f^	na
Moxidectin	24 µg/kg	0, 30, 60, 90	5 of 6	0, 1, 1, 1, 2, 3	1.1^g^	96.8
Moxidectin	24 µg/kg	0, 30, 60, 90, 120, 150	5 of 6	0, 1, 1, 2^*^, 2, 4	1.4^g^	96.1
Heartgard® Plus	Min. of 6 µg/kg^d^	0, 30, 60, 90, 120, 150	6 of 6	25, 25, 27, 27, 36, 36	29.0^f^	18.7
Interceptor® Plus	Min. of 500 µg/kg^e^	0, 30, 60, 90, 120, 150	6 of 6	16, 26, 28, 33, 34, 37	28.1^f^	21.2

^a^Each dog was inoculated with 50 infective ZoeLA L_3_ of *D. immitis* on Day -30, except for one dog (*) treated with six monthly doses of moxidectin which received 49 infective larvae

^b^Geometric mean counts with the same superscript letters (f-g) are not significantly different (*P* ≥ 0.05)

^c^All dogs were necropsied for recovery of adult *D. immitis* on Day 243 (273 days post-inoculation)

^d^Heartgard® Plus (ivermectin + pyrantel pamoate) administered according to commercial label directions which resulted in administration of 6.2–15.5 µg/kg of ivermectin

^e^Interceptor® Plus (milbemycin oxime + praziquantel) administered according to commercial label directions which resulted in administration of 0.5–1.2 mg/kg milbemycin oxime

All 6 negative control-treated dogs were infected with a geometric mean of 35.6 worms (range, 24–41). Five of 6 of the dogs treated with moxidectin for four monthly treatments were infected with a geometric mean of 1.1 worms (range, 0–3), and 5 of the 6 dogs treated with moxidectin for six monthly treatments were infected with a geometric mean of 1.4 worms (range 0–4). All 6 dogs treated with Heartgard® Plus were infected with a geometric mean of 29.0 worms (range, 25–36), and all 6 dogs treated with Interceptor® Plus were infected with a geometric mean of 28.1 worms (range, 16–37).

Geometric mean worm counts for both moxidectin groups were significantly lower than those for the negative control group (14.75 ≤ *t*_(8)_ ≤ 15.31, *P *< 0.0001), while geometric mean worm counts for the Heartgard® Plus and Interceptor® Plus groups were not significantly different from the negative control group (1.08 ≤ *t*_(8)_ ≤ 1.25, *P* ≥ 0.2471). There was no significant difference between the geometric mean worm counts for the two moxidectin groups (*t*_(*8*)_= 0.56, *P *= 0.5876); however, the geometric mean worm counts for both moxidectin groups were significantly lower from those for the groups treated with Heartgard® Plus and Interceptor® Plus (13.50 ≤ *t*_(8)_ ≤ 14.23, *P *< 0.0001), which were not significantly different from each other (*t*_(8)_= 0.17, *P *= 0.8709).

Preventive efficacies relative to negative control were 96.8% for the group treated with four monthly doses of moxidectin, 96.1% for the group treated with six monthly doses of moxidectin, 18.7% for the group treated with Heartgard® Plus, and 21.2% for the group treated with Interceptor® Plus.

### Efficacy against the ML-resistant JYD-34 strain

Blood microfilariae and adult *D. immitis* antigen results are summarized in Table 4. Adult *D. immitis* worm counts, efficacies relative to negative control, and statistical comparisons are summarized in Table 5.

**Table 4:** Adult *D. immitis* antigen and microfilariae test results (JYD-34 strain)

Treatment group	Oral dosage	Days of treatment	Individual	Day -30		Day 60		Day 180		Day 210		Day 236	
				Ag	MF	Ag	MF	Ag	MF	Ag	MF	Ag	MF
Negative control	na	0, 30, 60, 90, 120, 150	1	−	−	−	−	+	+	+	+	+	+
			2	−	−	−	−	+	+	+	+	+	+
			3	−	−	−	−	+	+	+	+	+	+
			4	−	−	−	−	+	+	+	+	+	+
			5	−	−	−	−	+	+	+	+	+	+
			6	−	−	−	−	+	+	+	+	+	+
Moxidectin	24 µg/kg	0, 30, 60, 90	1	−	−	−	−	−	−	+	−	+	−
			2	−	−	−	−	−	−	+	−	+	−
			3	−	−	−	−	−	−	−	−	−	−
			4	−	−	−	−	−	−	−	+	+	+
			5	−	−	−	−	−	−	+	−	+	−
			6	−	−	−	−	−	−	−	−	+	−
Moxidectin	24 µg/kg	0, 30, 60, 90, 120, 150	1	−	−	−	−	−	−	−	−	−	−
			2	−	−	−	−	−	−	−	−	−	−
			3	−	−	−	−	−	−	−	−	−	−
			4	−	−	−	−	−	−	−	−	−	−
			5	−	−	−	−	−	−	−	−	−	−
			6	−	−	−	−	−	−	+	−	+	−
Heartgard® Plus	Min of 6 µg/kg^a^	0, 30, 60, 90, 120, 150	1	−	−	−	−	+	+	+	+	+	+
			2	−	−	−	−	−	−	−	+	+	+
			3	−	−	−	−	+	+	+	+	+	+
			4	−	−	−	−	−	+	+	+	+	+
			5	−	−	−	−	+	+	+	+	+	+
			6	−	−	−	−	+	−	+	−	+	−
Interceptor® Plus	Min. of 500 µg/kg^b^	0, 30, 60, 90, 120, 150	1	−	−	−	−	+	+	+	+	+	+
			2	−	−	−	−	+	+	+	+	+	+
			3	−	−	−	−	+	+	+	+	+	+
			4	−	−	−	−	−	+	−	+	+	+
			5	−	−	−	−	+	+	+	+	+	+
			6	−	−	−	−	−	+	+	+	+	+

^a^Heartgard® Plus (ivermectin + pyrantel pamoate) administered according to commercial label directions which resulted in administration of 6.0–11.8 µg/kg of ivermectin

^b^Interceptor® Plus (milbemycin oxime + praziquantel) administered according to commercial label directions which resulted in administration of 0.5–1.0 mg/kg milbemycin oxime

*Abbreviations*: Ag, adult *D. immitis* antigen test; MF, *D. immitis* microfilariae test; −, negative; +, positive

**Table 5:** Efficacy of oral moxidectin (24 µg/kg) compared to Heartgard® Plus or Interceptor® Plus (JYD-34 strain)

Treatment	Oral dosage	Days of treatment	No. of infected dogs^a^	Adult *D. immitis* worm counts		Efficacy compared to negative control^c^
				Individual worm counts	Geometric mean^b^	% Reduction
Negative Control	NA	0, 30, 60, 90, 120, 150	6 of 6	30, 33, 33, 33, 35, 37	32.9^f^	na
Moxidectin	24 µg/kg	0, 30, 60, 90	5 of 6	0, 1, 1, 2, 2, 4	1.3^g^	95.9
Moxidectin	24 µg/kg	0, 30, 60, 90, 120, 150	2 of 6	0, 0, 0, 0, 1, 1	0.2^h^	99.3
Heartgard® Plus	min of 6 µg/kg^d^	0, 30, 60, 90, 120, 150	6 of 6	6, 9, 13, 14, 17, 18	11.9^i^	63.9
Interceptor® Plus	min. of 500 µg/kg^e^	0, 30, 60, 90, 120, 150	6 of 6	10, 13, 14, 16, 19, 22	14.9^i^	54.6

^a^Each dog was inoculated with 50 infective JYD-34 L_3_ of *D. immitis* on Day -30

^b^Geometric mean counts with the same superscript letters (f-i) are not significantly different (*P* ≥ 0.05)

^c^All dogs were necropsied for recovery of adult *D. immitis* on Day 237 (267 days post-inoculation)

^d^Heartgard® Plus (ivermectin + pyrantel pamoate) administered according to commercial label directions which resulted in administration of 6.0 to 11.8 µg/kg of ivermectin

^e^Interceptor® Plus (milbemycin oxime + praziquantel) administered according to commercial label directions which resulted in administration of 0.5 to 1.0 mg/kg milbemycin oxime

All 6 negative control-treated dogs were infected with a geometric mean of 32.9 worms (range, 30–37). Five of 6 of the dogs treated with moxidectin for four monthly treatments were infected with a geometric mean of 1.3 worms (range, 0–4), and 2 of the 6 dogs treated with moxidectin for six monthly treatments were infected with a geometric mean of 0.2 worms (range, 0–1). All 6 dogs treated with Heartgard® Plus were infected with a geometric mean of 11.9 worms (range, 6–18), and all 6 dogs treated with Interceptor® Plus were infected with a geometric mean of 14.9 worms (range, 10–22).

Geometric mean worm counts for all treated groups were significantly lower than those for the negative control group (6.90 ≤ *t*_(8)_ ≤ 21.46, *P* ≤ 0.0001). Geometric mean worm counts for the group treated with six monthly doses of moxidectin were significantly lower (*t*_(8)_= 2.35, *P *= 0.0470) than those for the group treated with four monthly doses, and the geometric mean worm counts for both moxidectin groups were significantly lower than those for the groups treated with Heartgard® Plus and Interceptor® Plus (6.50 ≤ *t*_(8)_ ≤ 14.29, *P* ≤ 0.0002), which were not significantly different from each other (*t*_(8)_= 1.28, *P *= 0.2370).

Preventive efficacies relative to negative control were 95.9% for the group treated with four monthly doses of moxidectin, 99.3% for the group treated with six monthly doses of moxidectin, 63.9% for the group treated with Heartgard® Plus, and 54.6% for the group treated with Interceptor® Plus.

## Discussion

Heartworm disease is a serious and potentially fatal condition for dogs. For this reason, the goal of preventative treatments is appropriately focused on achieving 100% preventive efficacy. Although many infected dogs show no clinical signs, pulmonary arterial disease occurs in every infected dog, and infections with higher worm burdens can result in more significant clinical disease [13]. Within days of reaching the caudal pulmonary arteries, the adult worms cause traumatic arterial endothelial damage, and the severity of damage is directly related to the duration of the infection and the worm burden [14]. Infections with higher worm burdens can result in pulmonary hypertension with tricuspid insufficiency, which ultimately leads to congestive heart failure [15]. Dogs with higher worm burdens are also at increased risk of caval syndrome, which is often fatal [1, 5].

A review of the reported efficacy data has shown moxidectin to be the most potent of the MLs in preventing the development of *D. immitis* in dogs [4]. Moxidectin generally has a longer elimination half-life and a larger volume of distribution than both ivermectin and milbemycin, which allow for a longer persistence and greater distribution of moxidectin in host tissues, including adipose tissue where it is active against migrating *D. immitis* larvae [6]. These attributes make moxidectin an attractive molecule to optimize in new heartworm preventative products in the face of emerging ML heartworm resistance.

Recent laboratory studies have demonstrated that a single oral dose of moxidectin at 24 µg/kg alone or in combination with sarolaner and pyrantel (Simparica Trio™; Zoetis) was 100% effective against two recently collected *D. immitis* field strains characterized as not resistant to MLs [16]. Similarly, a recent clinical field study showed that 11 monthly oral doses of moxidectin at 24 µg/kg in combination with sarolaner and pyrantel were 100% effective in preventing the development of heartworm disease in 246 client-owned dogs enrolled from heartworm-endemic regions of the USA, including the lower Mississippi River Valley (LMRV) where ML-resistance is of high concern [4], compared to the 98.3% efficacy provided in the 117 dogs treated with 11 monthly doses of Heartgard® Plus [16]. Of the two Heartgard® Plus-treated dogs that were positive for *D. immitis* antigen, one was also positive for *D. immitis* microfilariae. The presence of microfilaremia in this dog after receiving an ML for 11 consecutive months strongly supports that this dog was infected with a ML-resistant *D. immitis* strain [17, 18]. Another recent clinical field study [19] which was also conducted in heartworm-endemic regions of the USA, including the LMRV, showed that moxidectin in an extended-release injectable formulation (ProHeart® 12; Zoetis) provided 100% heartworm prevention in 236 dogs compared to the 98.8% efficacy provided in the 218 dogs treated monthly with Heartgard® Plus. Of the four Heartgard® Plus dogs that were positive for *D. immitis* antigen in that study, three were also positive for *D. immitis* microfilariae with confirmed monthly compliance; this also supports the likelihood that these dogs were infected with an ML-resistant strain of *D. immitis.*

**Fig. 1:**
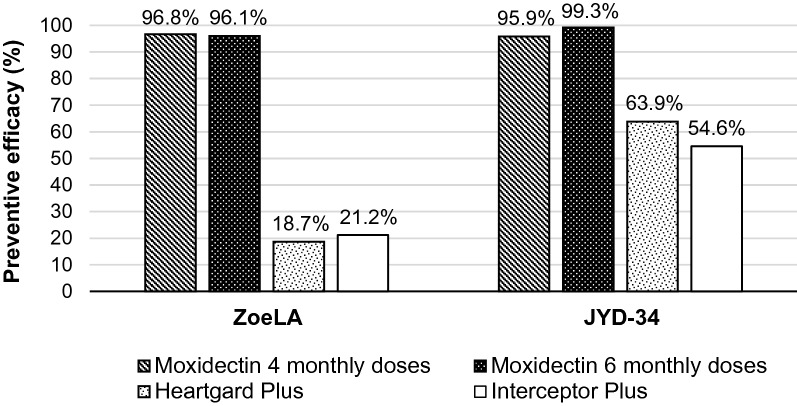
Efficacy of oral moxidectin (24 µg/kg) against ML-resistant heartworm strains compared to Heartgard® Plus or Interceptor® Plus

In the present studies against two known ML-resistant *D. immitis* strains, four or six monthly doses of 24 µg/kg moxidectin provided ≥ 95.9% efficacy relative to the negative control, compared to the ≤ 63.9% efficacy provided by Heartgard® Plus and the ≤ 54.6% efficacy provided by Interceptor® Plus (Fig. 1). Moxidectin at either four or six monthly doses also resulted in significantly fewer (6.50 ≤ *t*_(*8*)_ ≤ 14.29, *P* ≤ 0.0002) adult *D. immitis* in dogs compared to Heartgard® Plus or Interceptor® Plus against both ML-resistant strains. Treatment with moxidectin also resulted in more dogs being free of heartworms or having only low worm burdens. All 24 dogs treated with six monthly doses of Heartgard® Plus or Interceptor® Plus were infected with adult heartworms with a range of 6–37 worms. In contrast, moxidectin provided 100% preventive efficacy (no worms recovered at necropsy) in seven of the 24 dogs, and the remaining dogs had only between 1–4 worms. When extrapolating this activity to a large population, this difference in worm burden could translate to a substantial clinical benefit for those dogs treated with 24 µg/kg moxidectin compared to those treated with either Heartgard® Plus or Interceptor® Plus.

The preventive efficacies provided by moxidectin against the ML-resistant *D. immitis* strains evaluated in the present studies support the outcomes from previous studies [4] and a comparison of data from previous studies and the present studies are presented in Table 6. Against ZoeLA, three monthly doses of 24 µg/kg moxidectin resulted in significantly fewer adult worms (*t*_(35)_= 3.33, *P *< 0.0001) and 99.5% efficacy relative to placebo control. Against JYD-34, three monthly doses of 24 µg/kg moxidectin yielded significantly fewer adult worms (*t*_(3)_= 2.85, *P *< 0.0001) and 98.8% efficacy, while a single dose resulted in significantly fewer adult worms (*t*_(7.37)_= 4.92, *P *= 0.0015) but only 53.2% efficacy. In the study against the JYD-34 strain, six monthly doses of moxidectin resulted in significantly fewer (*t*_(8)_= 2.35, *P *= 0.0470) adult *D. immitis* than four monthly doses, providing further evidence that increasing the number of consecutive monthly doses improves the efficacy of moxidectin against ML-resistant *D. immitis.* The American Heartworm Society recommends continuous year-round administration of preventative drugs to ensure prevention of heartworm disease [5].

**Table 6:** Efficacy of oral moxidectin (24 µg/kg) against ML-resistant heartworm strains compared to Heartgard® Plus or Interceptor® Plus: summary

Oral treatment^a^	No. of monthly treatments^b^	ZoeLA				JYD-34			
		No. of infected dogs^c^	Adult *D. immitis* worm counts		Efficacy compared to negative control (%)	No. of infected dogs^c^	Adult *D. immitis* worm counts		Efficacy compared to negative control (%)
			Range	Geometric mean			Range	Geometric mean	
Moxidectin	1	Not tested				8 of 8	7–29	16.8	53.2^d^
Moxidectin	3	1 of 5	0–1	0.1	99.5^d^	1 of 5	0–2	0.2	98.8^d^
Moxidectin	4	5 of 6	0–3	1.1	96.8^e^	5 of 6	0–4	1.3	95.9^e^
Moxidectin	6	5 of 6	0–4	1.4	96.1^e^	2 of 6	0–1	0.2	99.3^e^
Heartgard® Plus	6	6 of 6	25–36	29.0	18.7^e^	6 of 6	6–18	11.9	63.9^e^
Interceptor® Plus	6	6 of 6	16–37	28.1	21.2^e^	6 of 6	10–22	14.9	54.6^e^

^a^Moxidectin administered at exact 24 µg/kg dosage. Heartgard® Plus and Interceptor® Plus administered according to commercial label directions which resulted in administration of 6.2–15.5 µg/kg of ivermectin and 0.5–1.2 mg/kg milbemycin oxime

^b^Treatment administration days: 1 monthly treatment: Day 0; 3 monthly treatments: Days 0, 28 and 56 (studies from data reference 1) or Days 0, 30, and 60 (studies from this paper); 4 monthly treatments: Days 0, 30, 60 and 90; 6 monthly treatments: Days 0, 30, 60, 90, 120 and 150

^c^Each dog inoculated with 50 *D. immitis* L_3_ on Day -30 with adult heartworm recovery at necropsy 133 to 273 days after L_3_ inoculation

^d^Data reference: McTier et al. [4]

^e^Data reference: this paper

In most cases MLs are highly effective in the prevention of heartworm disease. It is estimated that only 30% of dogs are on a ML preventative, and of those the most common cause for LOE is failure to regularly administer the product according to label directions. Although further work is needed to better understand their exact geographic distribution, the presence of multiple ML-resistant *D. immitis* strains in the field has been confirmed [4]. Collectively, data from the present laboratory studies and those from earlier laboratory and field studies support that oral moxidectin administered monthly at a minimum dosage of 24 µg/kg should provide robust heartworm prevention against a variety of *D. immitis* strains to which most dogs in the USA will likely be exposed, including those confirmed to be ML-resistant.

## Conclusions

In two laboratory studies designed to assess preventive efficacy against ML-resistant *D. immitis* strains, moxidectin administered at an oral dosage of 24 µg/kg for four or six months provided ≥ 95.9% reduction in ML-resistant adult *D. immitis* relative to negative controls, and resulted in significantly fewer ML-resistant adult *D. immitis* than did Heartgard® Plus or Interceptor® Plus administered for six months at their approved label dosages.

## Data Availability

Data supporting the conclusions of this article are included within the article.

## Abbreviations

HPMC: hydroxypropyl methycellulose
JYD-34: *Dirofilaria immitis* strain JYD-34
L_3_: third-stage larvae
LMRV: lower Mississippi River valley
LOE: lack of efficacy
ML: macrocyclic lactone
ZoeLA: *Dirofilaria immitis* strain ZoeLA-2013
